# Validation of antibodies for the specific detection of human TRPA1

**DOI:** 10.1038/s41598-019-55133-7

**Published:** 2019-12-06

**Authors:** H. S. Virk, M. Z. Rekas, M. S. Biddle, A. K. A. Wright, J. Sousa, C. A. Weston, L. Chachi, K. M. Roach, P. Bradding

**Affiliations:** Department of Respiratory Sciences, University of Leicester, UK Institute of Lung Health and NIHR Leicester BRC-Respiratory, Leicester, United Kingdom

**Keywords:** Ion channel signalling, Calcium signalling, Target validation, Ion channels in the nervous system, Immunoblotting

## Abstract

The transient receptor potential cation channel family member ankyrin 1 (TRPA1) is a potential target for several diseases, but detection of human TRPA1 (hTRPA1) protein in cells and tissues is problematic as rigorous antibody validation is lacking. We expressed hTRPA1 in a TRPA1-negative cell line to evaluate 5 commercially available antibodies by western blotting, immunofluorescence, immunocytochemistry and flow cytometry. The three most cited anti-TRPA1 antibodies lacked sensitivity and/or specificity, but two mouse monoclonal anti-TRPA1 antibodies detected hTRPA1 specifically in the above assays. This enabled the development of a flow cytometry assay, which demonstrated strong expression of TRPA1 in human lung myofibroblasts, human airway smooth muscle cells but not lung mast cells. The most cited anti-TRPA1 antibodies lack sensitivity and/or specificity for hTRPA1. We have identified two anti-TRPA1 antibodies which detect hTRPA1 specifically. Previously published data regarding human TRPA1 protein expression may need revisiting.

## Introduction

The transient receptor potential cation channel family member ankyrin 1 (TRPA1) is an ion channel with high Ca^2+^ permeability that is activated by numerous noxious stimuli and by multiple products of oxidative stress^1–4^. TRPA1 is a drug target with antagonists in phase I and II clinical trials^5,6^. It is considered a potential target in multiple pain conditions including neuropathic, inflammatory and migraine pain, in addition to cough sensitivity, airway inflammation and fibrosis^5,7–12^.

A well-recognised limitation in terms of studying protein expression is the lack of antibodies evaluated to agreed standards for validation^13^. The use of insufficiently validated antibodies may have contributed to important failures of reproducibility and translation, for example in breast cancer and asthma research^14,15^. This problem has also been recognised in the field of TRP channel research^16^. Validation data is often not provided with antibodies and usually does not exclude the possibility of significant recognition of other antigens, in addition to the antigen of interest. This may lead to unreliable results but despite this, antibody validation using stringent controls is often not presented in the literature. Genetic strategies for the production of positive and negative controls, and immunocapture mass spectroscopy are accepted as robust methods for the evaluation of antibody specificity^13^.

We wished to study the expression profile of TRPA1 in human lung and airway derived cells. In order to validate antibodies, we generated positive and negative controls using a dual promoter vector to co-express hTRPA1 with a GFP reporter in a cell line that does not contain detectable levels of endogenous hTRPA1. We used these cells to evaluate the 3 most commonly used anti-TRPA1 antibodies according to the antibody database CiteAb^17^; all of these are polyclonal rabbit (Table 1**)**. Two are raised against epitopes in hTRPA1, one (Ab58844) is raised against rat TRPA1 but is predicted to work with hTRPA1 by the manufacturer (Table 1). We also evaluated 2 lesser used monoclonal mouse antibodies.

**Table 1:** Anti-hTRPA1 antibodies studied.

Cat no	Clone	Lot numbers tested (WB)	Epitope region	Supplier	Species and reactivity	Conc µg/mL	Recommended applications	Cite Ab Citations
NB110-40763		E2 E3 F2	N-terminus Intracellular AA 1-100	Novus Biologicals	Polyclonal rabbit anti hTRPA1	2	FC/FACS, ICC-IF, IHC and WB	23
Ab58844		GR3208982-3	C-terminus Intracellular AA 1060-1075	AbCam	Polyclonal rabbit anti rat TRPA1 predicted to work with human	2	IHC	17
ACC- 037		AN1702 AN1202 AN1150	1st extracellular loop AA 747-760	Alomone Labs	Polyclonal rabbit anti hTRPA1	4.5	ICC, IHC, IP, and WB	11
sc-376495	C-5	G1718	C-terminus Intracellular AA 965-1119	Santa Cruz	Monoclonal mouse anti hTRPA1	0.125–1*	WB, IP, IF, ELISA	0
ST1685	6G8	H3131-6G8	C-terminus Intracellular AA 1033–1118	Merck	Monoclonal mouse anti hTRPA1	5	ELISA and WB	2

AA amino acids; FC/FACS flow cytometry; ICC immunocytochemistry; IF immunofluorescence; IHC immunohistochemistry; IP immunoprecipitation; WB western blotting. *0.125 µg/mL of primary conjugated antibody for flow cytometry in our work, 1 µg/mL for all other applications. Concentrations only refer to those used by us in this work.

## Results

**Figure 1:**
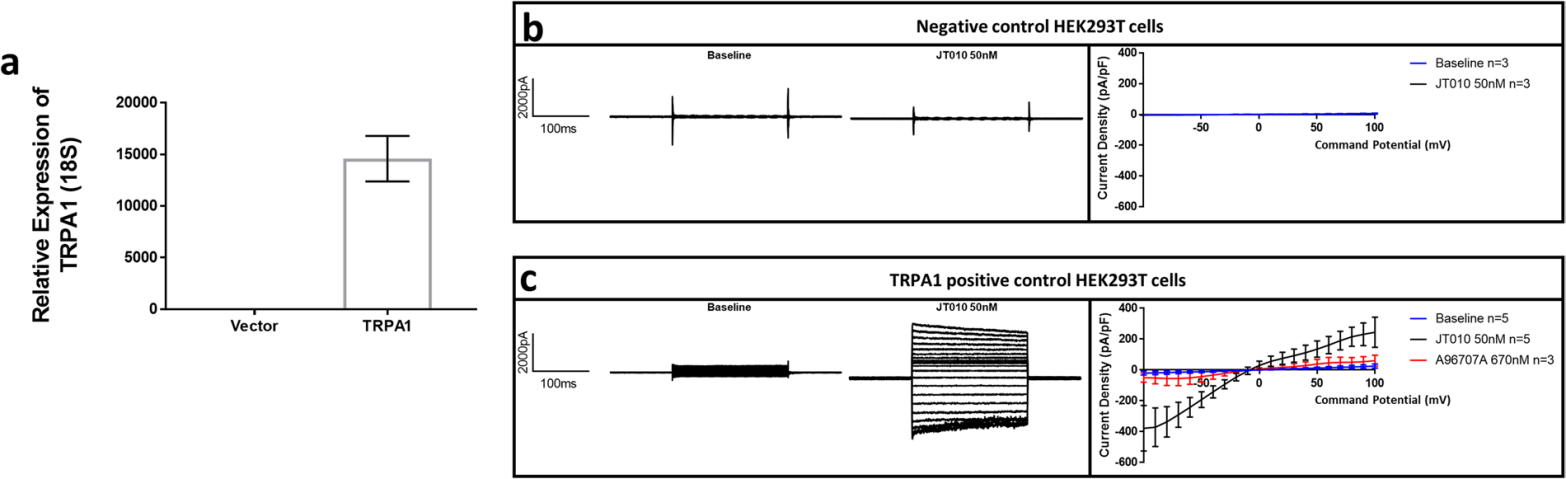
Validation of positive and negative control cells by qPCR and patch clamp electrophysiology. (**a**) Real time quantitative PCR of HEK293T cell RNA for TRPA1 mRNA. The negative control cells were transduced with an empty GFP vector (Vector), the positive control cells were transduced with dual promoter TRPA1 and GFP lentiviral vector (TRPA1), data presented are mean and standard error of mean of two independently generated cell lines. (**b**,**c)** Whole cell recordings by patch clamp electrophysiology of negative control cells (n = 3) or positive control cells (n = 5) stimulated with the specific TRPA1 agonist JT-010. GFP fluorescence was confirmed in all cells prior to recording. The left two panels show representative baseline and JT-010 stimulated currents. The right panel shows the mean current voltage relationship of all recordings, including following addition of the specific TRPA1 antagonist A967079, which was added when a JT-010 stimulated current was observed (only in positive controls). These experiments demonstrate low or no expression of TRPA1 mRNA in negative control cells, which corresponds to no functional response. The positive controls display high mRNA and strong functional responses.

Successful cloning of a dual promoter lentiviral TRPA1 and GFP expression vector was confirmed by sequencing of the insert. TRPA1 mRNA from transduced HEK293T cells were >100 000 fold higher in the positive than the negative control cells, where it was close to the limit of detection (Fig. 1a.) Whole cell currents consistent with TRPA1 were not observed in negative control cells (Fig. 1b), positive control cells displayed large TRPA1 currents (Fig. 1c).

**Figure 2:**
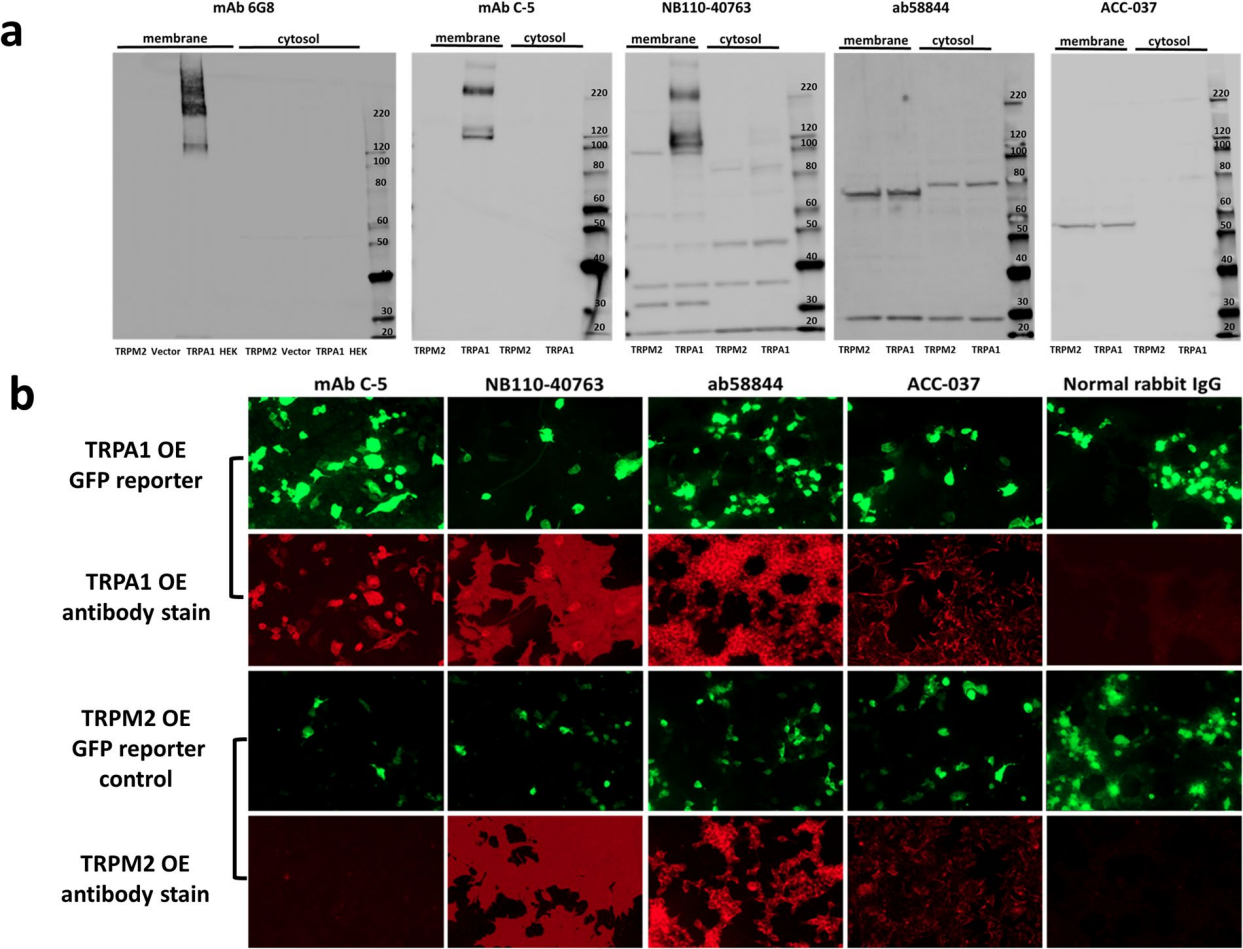
Evaluation of anti-TRPA1 antibody specificity by western blotting and immunofluorescence. (**a**) Lysates derived from HEK293T cells transiently transfected with the TRPA1 and GFP expression construct (TRPA1 OE) or TRPM2 and GFP negative control (TRPM2 OE) were probed with the antibodies indicated. Additional untransfected and GFP only controls are presented in the first blot (6G8). Uncropped full length blots are displayed, gaps between the blots are present to indicate the use of a different antibody. Mouse mAbs C-5 and 6G8, and NB110-40763 can detect TRPA1 only in the membrane fraction of lysates at the expected molecular weight (127.5 kDa). Several TRPA1-specific bands are observed above this weight. NB110-40763 also detects several other antigens. Ab58844 and ACC- 037 only appear to detect antigens other than TRPA1 in the conditions used. (**b**) The performance of the same antibodies was evaluated by immunofluorescence (red) in comparison to the expression of the GFP reporter (green), or control TRPM2 and GFP overexpressing cells as for western blotting. Mouse mAb C-5 again shows high specificity with a strong correlation between the expression of the GFP reporter and antibody staining, and no staining in TRPM2 and GFP overexpressing controls. NB110-40763 also demonstrates some sensitivity but has high background that likely does not correspond to Fc binding of the antibody, as the other two rabbit polyclonal antibodies or isotype control did show different staining patterns. Ab58844 and ACC- 037 only appear to detect antigens other than TRPA1 in the conditions used.

HEK293T cells transfected with the TRPA1 construct (validated in Fig. 1) were then used to generate lysates for western blotting (Fig. 2a) and cells for immunofluorescent staining (Fig. 2b). Controls with no transfection, empty vector, or overexpression of a different gene (TRPM2) in the same vector were used. The use of a different gene helped ensure equal transfection efficiency and allowed us to detect possible cross reactivity with other genes from the same family.

The performance of 5 antibodies (Table 1) was evaluated by western blotting (Fig. 2a). The 2 mouse monoclonal antibodies (mAbs) (Table 1) detected TRPA1 at the expected molecular weight (127.5 kDa). There are also additional bands detected at higher molecular weights that are TRPA1-specific given that all controls were negative. These mouse mAbs are therefore specific for TRPA1 in western blotting (Fig. 2a). NB110-40763 also detected TRPA1 at the expected size. However, this antibody appeared to label several other proteins, including one at 100 kDA which could potentially be misidentified as TRPA1 without reference to robust positive and negative controls. Ab58844 and ACC- 037 only appear to detect antigens other than hTRPA1 in the conditions used (Fig. 2a). Ab58844 is raised against a non-human TRPA1 and is only predicted to be able to detect hTRPA1 (see Table 1).

Similarly, by immunofluorescent staining of methanol fixed cells overexpressing TRPA1 and a GFP reporter, mAb C-5 detected TRPA1 in overexpressing cells (as confirmed by the GFP reporter) (Fig. 2b). NB110-40763 was also able to detect TRPA1 matching the GFP reporter, but there was also strong additional staining (Fig. 2b). The additional staining pattern observed with NB110-40763 was different to the other 2 polyclonal rabbit antibodies used and to isotype staining (Fig. 2b). This suggests it was mediated by the antigen binding fragment of the antibody binding to unknown antigens, consistent with the western blot result (Fig. 2a). Ab58844 and ACC-037 were only able to detect proteins other than hTRPA1 in the conditions used.

ACC- 037 only detected a single band in western blot that was not TRPA1 in the HEK293T cells studied. The sequence of the immunising peptide has significant homology to human vimentin as shown in the supplementary information (Fig. s1). We therefore performed immunocapture mass spectroscopy on the only band detected in the western blots with vimentin expressing cells (human lung myofibroblasts and human lung mast cells). Vimentin was the most abundant non keratin detected in all samples. In cells expressing vimentin and TRPA1 (human lung myofibroblasts), only vimentin was detected by western blotting as shown in the supplementary information (Fig. s1). That this antibody recognises the immunising peptide epitope was confirmed by preadsorption of the antibody with the peptide, this blocked all antibody staining in western blotting as shown in the supplementary information (Fig. s2).

**Figure 3:**
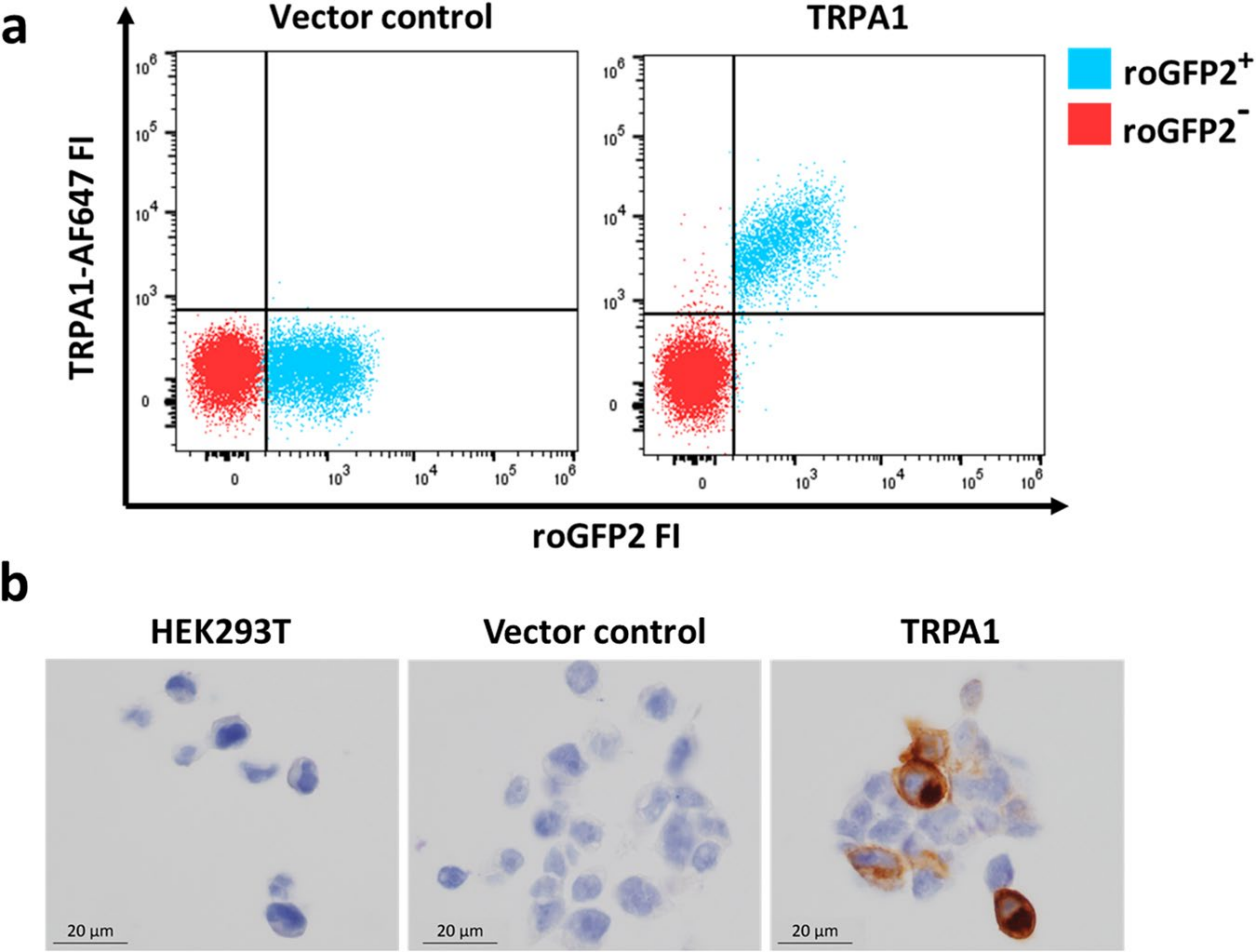
Validation of flow cytometry and immunocytochemistry hTRPA1 assays using mouse mAb C-5. (**a**) Dot plot of transiently transfected live HEK293(T) cells stained with Alexa Flour 647 (AF647) primary conjugated mouse mAb C-5. Cells were transfected with a TRPA1 overexpression construct with a roGFP2 reporter or a control roGFP2 vector. Only cells transfected with TRPA1 stained and the intensity of expression of the reporter correlated with TRPA1 staining intensity. (**b**) Paraffin embedded cell pellets of untransfected HEK293T cells, transiently transfected HEK293T cells (vector control and TRPA1) stained with mouse mAb C-5. Further uncropped images with more cells are presented in the supplemental information (Fig. s3).

We then selected the hTRPA1 specific mAB C-5 in order to optimise detection of hTRPA1 by flow cytometry. Fixed and permeabilised control cells were stained with mAB C-5 conjugated to AlexaFluor™ 647 and analysed by flow cytometry (Fig. 3a). Because fixing and permeabilising substantially diminished the brightness of the original GFP reporter, a new construct was cloned using an alternative GFP protein, roGFP2. Once dead cells were gated out using Zombie Aqua dead cell stain, TRPA1 signal was only detected in roGFP2 reporter positive controls, but not in untransfected or negative control transfected cells (Fig. 3a). The intensity of the TRPA1 staining corresponded to the intensity of the roGFP2 reporter (Fig. 3a).

Vector control and hTRPA1 transfected HEK293T cell pellets fixed and then embedded into wax were utilised to test the specificity of mAb C-5 by immunocytochemistry (Fig. 3b). A high pH antigen retrieval protocol enabled specific detection of TRPA1 by immunocytochemistry (Fig. 3b). Further uncropped images with more cells are presented in the supplemental information (Fig. s3). The transfection efficiency for this experiment is approximately 40%, which explains why not all cells stain for TRPA1. Unfortunately, despite using similar antigen retrieval strategies on paraffin embedded human lung tissue containing cells known to express TRPA1, the mAbs C-5 and 6G8 showed no staining.

**Figure 4:**
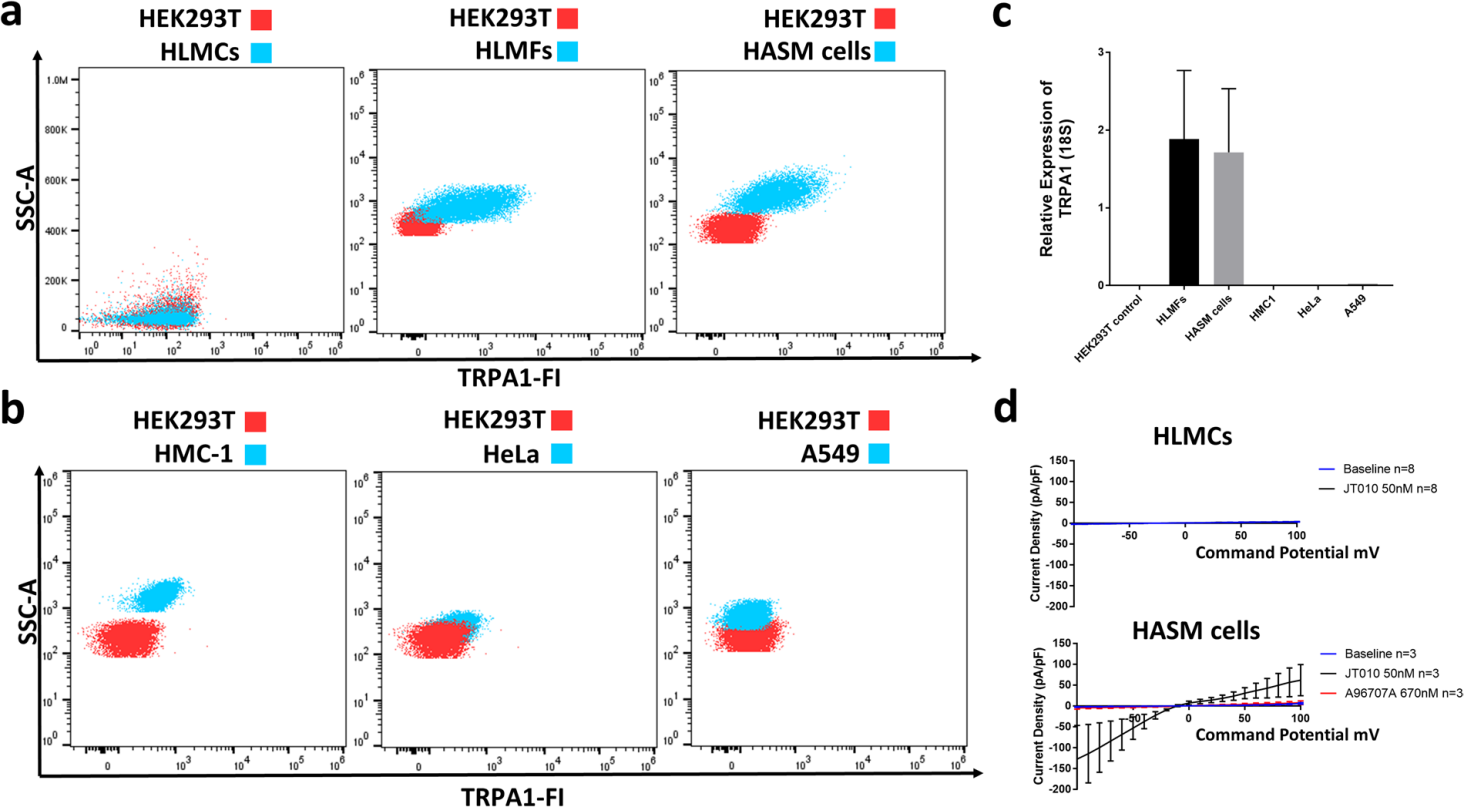
Expression profiling of TRPA1 in human lung and airway derived cells and cell lines. Flow cytometry comparing hTRPA1 staining between negative control cell line HEK293T and (**a**) human lung mast cells (HLMCs, no staining observed, representative of 4 donors), human lung myofibroblasts (HLMFs, staining observed in 4 out of 4 donors) and human airway smooth muscle cells (HASM, staining observed in 7 out of 7 donors). (**b**) HMC-1 cell line, HeLa cell line and A549 cell line. (**c**) expression of mRNA for TRPA1 pattern matches expression pattern observed by flow cytometry in HLMF, HASM and cell lines. (**d**) Mean current voltage relationships of whole cell currents by patch clamp electrophysiology show TRPA1 currents in HASM cells (3 cells from 3 donors) but not HLMCs (representative of 8 cells in 2 donors). Example raw currents are display in the supplemental information (Fig. s5).

Utilising the mAb C-5 that we validated by flow cytometry in HEK293T cells (Fig. 3a**)**, we set out to detect TRPA1 on human lung and airway derived cells. Since we knew that untransfected HEK293T cells do not express functional TRPA1 we mixed them with human lung mast cells to create an internal, biological negative control. TRPA1 staining was not observed in human lung mast cells (HLMCs) from 4 different donors (Fig. 4a) where the identity of isolated cells was confirmed by co-expression of CD45 and CD117. The gating strategy used in these mixtures is shown in the supplementary information (Fig. s4). Human lung myofibroblasts (HLMFs, Fig. 4a) and human airway smooth muscle (HASM, Fig. 4a) showed strong staining when compared to untransfected HEK293T cells stained in the same way in a separate tube.

We also examined the human mast cell line HMC-1, the cervical cancer cell line HeLa and the lung cancer cell line A549 similarly (Fig. 4b), very weak or no staining was observed. These results were corroborated by qPCR showing only relatively high mRNA in positively stained cell types (HLMFs and HASM cells, Fig. 4c). A549 cells did show TRPA1 mRNA expression higher than the negative control cell line but was very low in comparison to HLMFs and HASM. HASM cells and HLMCs were also tested for functional responses by whole cell recording by patch clamp electrophysiology – consistent with the flow cytometry, functional responses were only observed in HASM cells (Fig. 4d and supplementary information Fig. s5). The results of the flow cytometry TRPA1 protein expression experiments were therefore consistent with the results of qPCR and patch clamp electrophysiology.

## Discussion

TRPA1 is a promising therapeutic target with phase I and II clinical trials for antagonists underway in cough and neuropathic pain^5,6^. Much of our knowledge about expression of TRPA1 protein in human cells and tissue has relied on antibodies where validation of specificity was not undertaken or not presented. Antibody specificity is a major problem in research^13–16^. Our data show major discrepancies between antibodies used for the detection of hTRPA1, both with their ability to detect hTRPA1 but also their specificity for hTRPA1. Data using TRPA1 knockout mice has been partially presented that suggests two of the antibodies tested have specificity when used in mouse^18,19^, however our data shows these findings do not apply to human TRPA1. We have used our well validated tools to produce data that challenges what was believed to be known about expression of TRPA1 protein in human cells and tissues.

By using overexpression of hTRPA1 with GFP reporters in an hTRPA1-negative cell line to create robust positive and negative controls that are otherwise identical, we are confident in the data generated. Our results suggest that in the tested applications and conditions, the most commonly used anti-hTRPA1 antibodies, which are all rabbit polyclonals, lack both specificity for human TRPA1 and in two cases, the ability to detect hTRPA1 in this system. In contrast, two infrequently used mouse mAbs which detect similar epitopes on the C-terminus of hTRPA1, have clear specificity for hTRPA1 in hTRPA1-transfected HEK293T cells. mAb C-5 was able to detect hTRPA1 by immunofluorescence, immunocytochemistry and flow cytometry where it continued to demonstrate specificity.

One of the polyclonal rabbit antibodies did have sensitivity for hTRPA1 by both western blotting and immunofluorescence. However, it also detected numerous other proteins in western blotting, likely via its antigen binding fragment, as the additional antigens detected were not detected by any other polyclonal rabbit, or by an isotype control in immunofluorescence. This antibody can still be used for western blotting, but it is important to present a full western blot and show biological negative controls, as a band close to the correct molecular weight to hTRPA1, that is not specific to hTRPA1, is observed in some cell types. When used in other applications it may be impossible to determine whether any observed differences are because of a difference in TRPA1 expression, or in one of the other antigens that are detected by this antibody. One of the other polyclonal rabbit antibodies demonstrated strong reactivity for human vimentin and did not appear to recognise hTRPA1 in any of the tested applications in the conditions used.

Having optimised the detection of hTRPA1 protein using C-5 mouse mAb by flow cytometry, we tested the expression of TRPA1 protein in primary human airway/lung cell types and cell lines. We found no evidence of expression of TRPA1 in CD45+ CD117+ human lung mast cells. Previous work has suggested expression of TRPA1 by other mast cells^20,21^, including those from other species. We note that this work^20,21^ was completed using NB110-40763 which is not specific for TRPA1. However significant mast cell heterogeneity is well described^22–24^, and in light of our findings further work is needed to confirm previous observations in the field with the better performing mAbs. Similarly, TRPA1 protein was reportedly detected by NB110-40763 antibody in the cancer cell line A549^25^. In our work we have not seen evidence for TRPA1 protein expression in A549 cells using a robustly validated antibody. Our qPCR data shows good correspondence with our flow cytometry and patch clamp electrophysiology data across different cell types. TRPA1 staining was only observed in cell types with functional responses, in these cell types mRNA expression was many fold higher than the negative control HEK293T cells.

Strong staining for TRPA1 using flow cytometry was observed in primary human lung myofibroblasts, human airway smooth muscle cells. This is consistent with previous work^18,26^. Unfortunately, neither C-5 nor 6G8 were able to detect endogenous levels of TRPA1 expression in human tissues containing cells known to express TRPA1 by immunohistochemistry. However, flow cytometry may offer a way forward in terms of studying endogenous levels of hTRPA1 protein expression from fresh digested tissue.

Our work highlights the importance of undertaking relatively simple steps to validate the specificity of antibodies used for research, in this case for TRPA1. This would be expected to result in significant savings in time and resources for researchers in the field and ensure that research data are robust and reproducible. We encourage researchers and journals to make this type of data widely available for the benefit of the whole scientific community.

## Methods

### Production of overexpression constructs for TRPA1 with GFP reporters and negative controls

The pCDH-CMV-MCS-EF1-copGFP is a lentiviral mammalian expression vector with two mammalian promoters. The aim of our cloning was to generate a construct able to induce the expression of TRPA1 under the control of the CMV promotor and a green fluorescence protein under the control of the EF1α promoter. Mammalian Genome Collection sequence verified clone of human TRPA1 was purchased from Source Bioscience (IMAGE:100015422, MGC:183030 Accession: BC148423.1). Oligonucleotide primers were designed in order to generate a PCR product with the coding sequence of TRPA1, bound by restriction sites (XbaI and NotI) appropriate for directional cloning into the multiple cloning site of pCDH-CMV-MCS-EF1-copGFP.

Forward TRPA1 Xba1

ggg*tctaga*ATGAAGCGCAGCCTGAGGAAG

Reverse TRPA1 NotI

ggg*gcggccgc*ttaAGGCTCAAGATGGTGTGTTTTTGCC

This PCR product was sub-cloned into the pCR^™^4-TOPO^™^ vector for sequencing and cloning using the TOPO^™^ TA Cloning^™^ Kit. Sequence verified plasmid DNA from the subclone, and from the empty pCDH-CMV-MCS-EF1-copGFP vector was double digested with the restriction enzymes XbaI and NotI. Purified TRPA1 insert and the linearized lentiviral vector were then ligated using T4 DNA Ligase from Thermo Scientific^™^ according to the manufacturer’s instructions. The ligation product was then transformed into One Shot® Stbl3™ Chemically Competent E. coli and isolated plasmid DNA sequence verified.

NeBuilder online assembly tool was used to design primers for the replacement of copGFP with roGFP2-ORPI for the flow cytometry experiments. All genes of interest were fully sequenced in all vectors and the functionality of the viral particles was confirmed by transducing HeLa and analysing by flow cytometry for GFP.

### Sanger sequencing and sequence alignments

Sanger sequencing reactions of plasmid DNA were performed by GATC™. The sequence of the TRPA1 insert into the plasmids was confirmed. Alignment of the sequencing data with the reference sequence was performed using the online MultiAlin tool^27^.

### RT-qPCR for TRPA1

For RT-qPCR experiments cDNA was first synthesised based on a standard quantity of mRNA using the SuperScript™ VILO cDNA synthesis kit. Taqman® gene expression assays were then used according to the manufacturer’s instructions with primer/probes for TRPA1 (Hs00929065_M1) and 18 S (Hs9999901_s1 endogenous control) with the QuantStudio 5 Real-Time PCR system for relative gene expression by the delta-delta CT method.

### Transient transfection and production of lentiviral particles

Calcium phosphate co-transfection of these plasmids with 2nd generation lentiviral packaging vectors in HEK293(T) cells was used to produce viral particles. Briefly cells were plated in 10 cm dishes at 2 × 10^6^ cells per well in filtered DMEM media with 10% foetal calf serum and antimicrobials. The following day 20 µg of construct DNA together with 15 µg psPAX2 (packaging plasmid) and 5 µg of pMD2G (glycoprotein of the vesicular stomatitis virus (VSV-G) envelope expressing plasmid) was combined with CaCl_2_ to a final concentration of 0.25 M CaCl_2_. 500 µL of this solution was then added dropwise to bubbling HEPES buffered saline. The resulting mixture was allowed to stand for 30 minutes prior to addition to cell culture vessel. The cells were incubated with the DNA precipitate mixture overnight. The following day the media was replaced. Cell supernatant containing virus was harvested at 48 hours following addition of DNA precipitates and again at 72 hours. Viral supernatant was concentrated using PEGit™ viral precipitation solution. Transduction of 5 × 10^4^ HeLa cells was used to determine viral titre in HeLa TU/mL by measuring percentage GFP positive percentage by flow cytometry.

### Patch clamp electrophysiology

Single cell recordings from individual HEK293 (T), HASM or HLMC cells were performed using the whole cell variant of the patch clamp technique as previously described^28,29^. Standard external bath solution based on 140 mM sodium chloride and standard internal dialysing solution based on 100 mM potassium chloride containing 200 nM free ionic calcium was used. The potent and selective TRPA1 agonist JT-010^30^ was used to activate TRPA1, and the specific TRPA1 antagonist A967079 was used to further confirm the specificity of any observed currents.

### Western blotting

Membrane and membrane associated proteins were isolated separately from cytosolic proteins using the Mem-PER™ Plus Protein Extraction Kit from ThermoFisher® scientific according to the manufacturer’s instructions. Protein concentrations were quantified using the colorimetric Bio-Rad DC Protein Assay. Western blotting was performed using equal total protein for membrane lysates and separately for cytosolic lysates. Western blotting was performed with tris-acetate gel chemistry. Cell lysates were loaded in reducing conditions into precast 7% NuPage® Tris-acetate gels (Invitrogen) for separation by electrophoresis. Proteins were then transferred to a PVDF membrane using the Biorad Trans Blot Turbo Transfer System. Primary antibodies are described in Table 1. Secondary antibody goat anti-rabbit IgG-HRP from Santa Cruz were used with rabbit primary antibodies and goat anti-mouse IgG -HRP from Dako with mouse primary antibodies.

### Immunofluorescence

Transiently transfected HEK293T cells were methanol fixed and stained with primary antibodies using the same protocol as previously described^31^. A goat anti-rabbit APC conjugated secondary antibody (catalog A-10931 - Thermofisher Scientific) was used with rabbit polyclonals. Mouse mAb 1 (C-5) was used in a primary conjugated format as described above.

### Immunocytochemistry

Transiently transfected HEK293T cells were pelleted and fixed with 4% paraformaldehyde. The cell pellets were then mixed with liquid Histogel™ and then allowed to set. The Histogel pellet was then processed in paraffin.

Four micron sections were stained with antibody against TRPA1 (C-5) (1 µg/ml) or appropriate isotype control, EnVision™ FLEX + HRP kit from Agilent (K8002) was used, the antigens were retrieved in EnVision Flex Target Retrieval Solution (pH 9.0) at 97 °C. EnVision FLEX/HRP detection reagent was used as a detection system, colour reaction developed with DAB reagent as recommended by the manufacturer. All immunostained sections were counterstained with hematoxylin, dehydrated and mounted. Stained sections were photographed using a Zeiss Microscope with a x400 objective.

### Flow cytometry

Cells were harvested using a genetically modified collagenase with EDTA (TrypLE GIBCO). The cells were then stained using Zombie Aqua dead cell stain (Biolegend) for 15 minutes at room temperature. Cells were then fixed and permeabilised using eBioscience IC fixation permeabilisation buffers according to the manufacturer’s protocol (protocol A). 100 microlitres of cell suspension in permeabilisation buffer were stained with a primary Alexa Fluor 647 conjugated mouse mAb 1 (C-5) at 0.125 µg/mL for 20 minutes at room temperature. Following staining cells were washed in permeabilisation buffer and then resuspended in flow cytometry analysis buffer (2% fetal calf serum in phosphate buffered saline without Ca^2+^ or Mg^2+^). Cells were then analysed on an Attune NxT flow cytometer equipped with violet (405 nm), blue (488 nm) and yellow (566 nm) and red (637 nm) lasers. The expression of surface markers of mature mast cells, CD45 PerCP-eFlour710 (eBioscience Cat no: 46-0459-42) and CD117 PE-Dazzle 594 (Biolegend Cat no: 313225) was used to characterise HLMC cultures with TRPA1 staining as described above.

### Immunocapture mass spectroscopy

Lysates of HLMFs and HLMCs were incubated with ACC- 037 (Alomone Labs) conjugated to agarose beads. SDS-PAGE gel electrophoresis was performed on the precipitated proteins. The gel was then stained with Brilliant Blue G-Colloidal Concentrate according to the manufacturer’s instructions. All visible bands were cut out and sent for sequencing at the University of Leicester Protein Nucleic Acid Chemistry Laboratory using liquid chromatography-mass spectroscopy/mass spectroscopy (LC-MS/MS).

### Cell culture (HEK293T, HeLa, HASM, HLMF, HLMC, A549)

All cell cultures were maintained at 37 °C with 5% CO_2_ and 95% air.

HEK293 (T) cells and A549 cells were grown in T75 flasks with Dulbecco’s Modified Eagle Media containing GlutaMAX^TM^, 4.5 g/L glucose) and supplemented with 10% fetal bovine serum (FBS) and 1% antibiotic/antimycotic. HeLa cells were grown in T75 flasks in Minimum Essential Media supplemented with 10% FBS, 1% penicillin/streptomycin, and glutamine.

Non-fibrotic control (NFC) myofibroblasts were derived from healthy areas of lung from patients undergoing lung resection for carcinoma at Glenfield Hospital. All patients gave written informed consent, and the use of the tissue was approved by the National Research Ethics Service (references 10/H0402/12 and 07/MRE08/42). All methods were performed in accordance with the relevant guidelines and regulations. HLMF cultures were grown in T75 flasks in Dulbecco’s Modified Eagle Media containing Glutamax™ and 4.5 g/L glucose supplemented with 10% FBS, 1% antibiotic/antimycotic and 1% MEM Non-Essential Amino Acids Solution. The derivation and culture of HLMC and HASM cells was performed as previously described^32,33^. All participants donating tissue from which these primary cell cultures were derived gave written informed consent, and the collection of tissue was approved by the National Research Ethics Service. Reference numbers: 07/MRE08/42, 04/Q2502/74 and 08/H0406/189.

## Supplementary information

Supplementary information and figures

## Data Availability

All data generated during this study are either published in the main text or the supplementary information. The plasmids generated for this work are available upon request from the corresponding author.
