# Supplementary material for: Validation of antibodies for the specific detection of human TRPA1
Source: Sci Rep. 2019 Dec 6;9:18500. doi: 10.1038/s41598-019-55133-7 (PMC6898672; doi:10.1038/s41598-019-55133-7)
Supplement: Supplementary file 1 — Supplementary information and figures [file 41598_2019_55133_MOESM1_ESM.pdf]

Validation of antibodies for the specific detection of human TRPA1

HS Virk*, MZ Rekas, MS Biddle, AKA Wright, J Sousa, CA Weston, L Chachi, KM Roach, P Bradding

NIHR Respiratory BRC, Department of Respiratory Sciences, University of Leicester.

*Corresponding author: HSV hsv4@leicester.ac.uk

Supplementary information

Figure s1. Detection of human vimentin by ACC- 037 (Alomone Labs)

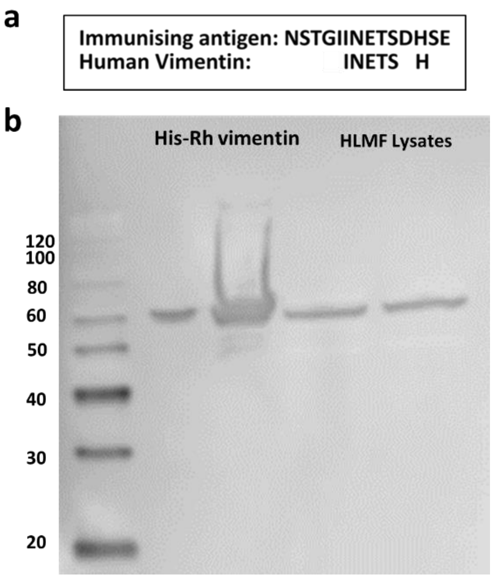

a There is some sequence homology between the immunising antigen used to generate polyclonal rabbit antibody ACC- 037 and human vimentin amino acid sequence. **b** His-tagged recombinant human (His-Rh) vimentin (2 concentrations) is detected by ACC- 037, a similar band is detected by this antibody in human lung myofibroblast (HLMF) lysates (2 donors).

Figure s2 Preadsorption of ACC-037 anti-TRPA1 antibody with the immunising peptide blocks staining by immunoblotting

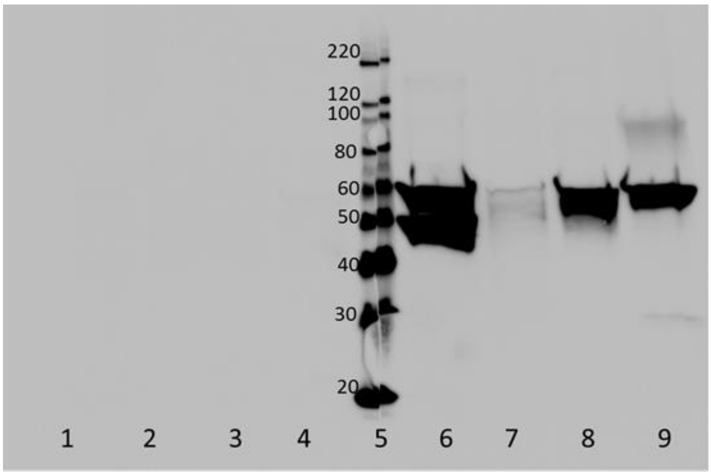

Lanes 1 and 9, human lung myofibroblast lyates, lanes 2,3,4,6 and 7 human lung mast cell lysates. Lanes 1-4 were preadsorped with the immunising peptide.

This shows that although ACC-037 stains antigens other than TRPA1, including vimentin, the staining is mediated by the antigen recognising binding of portion of the antibody.

Figure s3. Uncropped images of cell pellets from figure 3.

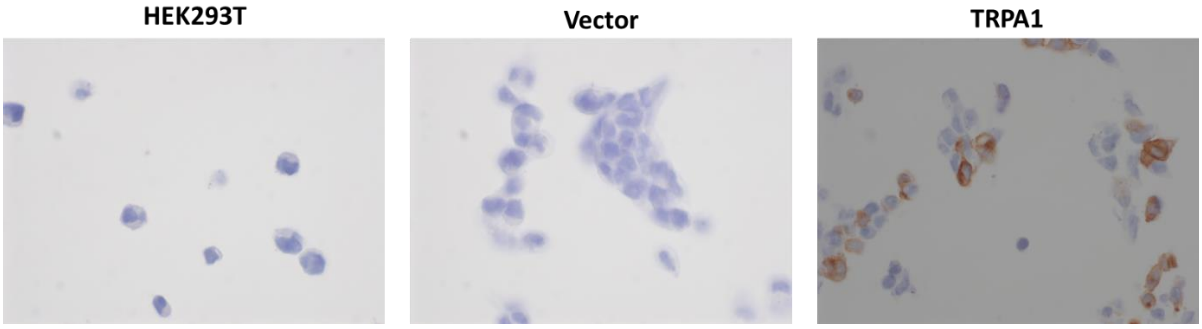

Figure s4. Validation of gating strategy of HEK293T and human lung mast cell (HLMC) mixtures

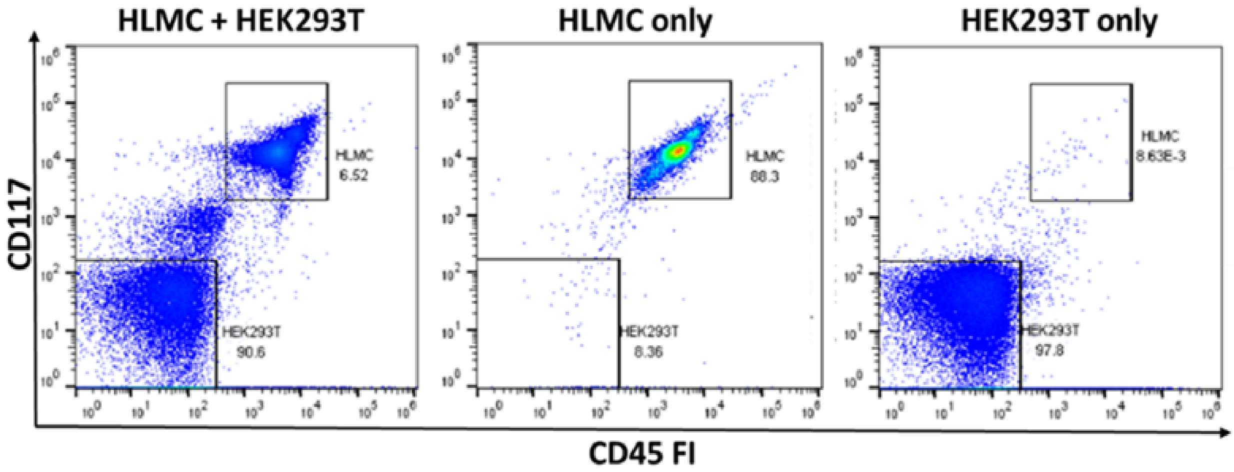

Flow cytometry of TRPA1-negative HEK293T cells mixed with human lung mast cells (HLMC + HEK293T) or mast cells alone (HLMC only) or HEK293T only. This shows that HEK293T cells can be identified as CD45 and CD117 negative with reference to mast cells.

Figure s5. Whole cell recordings from single human lung mast cell (HLMC) and human airway smooth muscle cell (HASM)

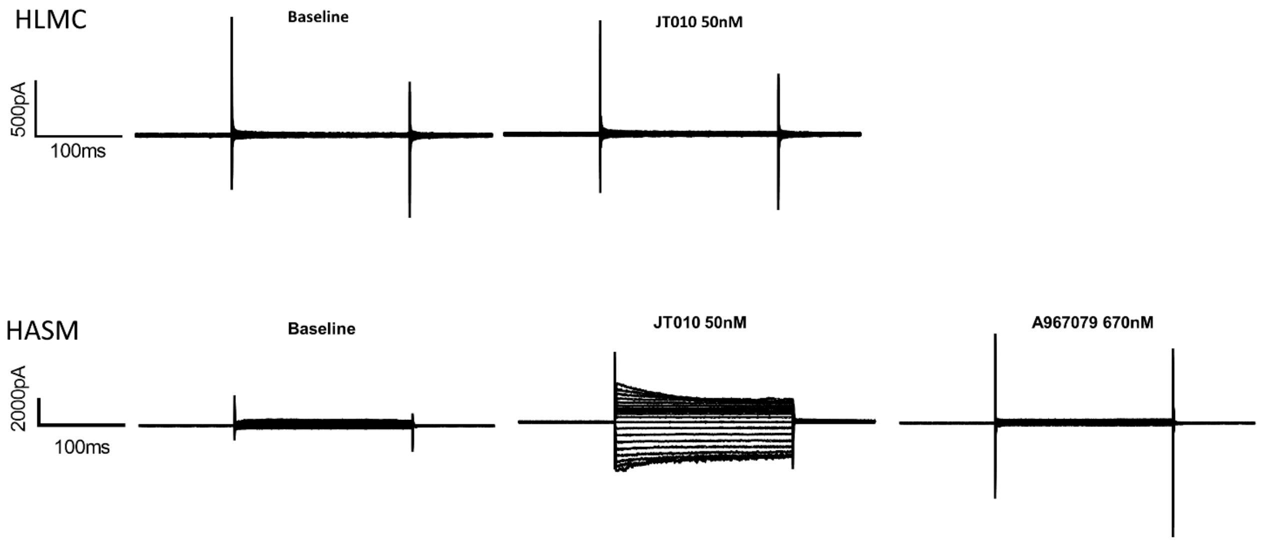

Representative raw currents from single cells derived using a voltage step protocol from a HASM cell and a HLMC
